# Malnutrition and its associated factors: a cross-sectional study with children under 2 years in a suburban area in Angola

**DOI:** 10.1186/s12889-019-6543-5

**Published:** 2019-02-21

**Authors:** João B. Humbwavali, Camila Giugliani, Luciana N. Nunes, Susana V. Dalcastagnê, Bruce B. Duncan

**Affiliations:** 1 Superior Institute of Health Sciences (ISCISA), Agostinho Neto University, Avenida 4 de Fevereiro, 77 Luanda, Angola; 2 Post-Graduation Program in Epidemiology, Federal University of Rio Grande do Sul (UFRGS), Rua Ramiro Barcellos, 2400/2° andar, CEP, Porto Alegre, RS 90035-003 Brazil

**Keywords:** Malnutrition, Infant nutrition disorders, Angola, Africa

## Abstract

**Background:**

The prevalence of child malnutrition in Angola is still very high, and little is known about its associated factors. The aim of this study was to identify these factors in children under 2 years in a suburban area of the country’s capital city.

**Methods:**

We used data from a cross-sectional population-based study conducted in 2010. The outcomes studied were stunting and underweight. Multivariable analysis was conducted; prevalence ratios were estimated by Poisson regression with robust variance using a hierarchical model.

**Results:**

Of the children studied (*N* = 749), 232 [32.0% (95% CI: 28.7–35.5%)] were stunted and 109 [15.1% (95% CI: 12.6–17.9%)] were underweight. In multivariable analysis, occurrence of diarrhea (PR 1.39 [95% CI: 1.07–1.87]) and the death of other children in the household (PR 1.52 [95% CI: 1.01–2,29]) were associated with stunting and underweight, respectively. In the model composed only of distal and intermediate factors, the primary caregiver not being the mother increased the prevalence of stunting by 42% (PR 1.42 [95% CI: 1.10–1.84], and a mother’s working outside the house while not being self-employed was associated with its reduced prevalence (PR 0.55 [95% CI: 0.34–0.89]). In the intermediate model, each additional month of delay in the onset of prenatal care increased the relative prevalence of underweight by 20% (PR 1.20 [95% CI: 1.03–1.40]).

**Conclusions:**

Despite the high prevalence rates of stunting and underweight, relatively few risk factors were identified for these conditions, suggesting that collective exposures are likely to play a major role in causing malnutrition in Angola. The individual factors identified can be useful for the development of strategies to deal with this public health problem.

**Electronic supplementary material:**

The online version of this article (10.1186/s12889-019-6543-5) contains supplementary material, which is available to authorized users.

## Background

It is known that good nutrition is a key driver in achieving a satisfactory level of human development. The World Health Organization (WHO) estimated in a recent report that there are 178 million undernourished children in the world, 20 million of whom suffer from severe malnutrition; undernutrition contributing to 3.5 to 5 million annual deaths among children under 5 years [1].

The monitoring of the goals against hunger set by the Millennium Development Goals ended in 2015 with the goals not having been met [2]. In Sub-Saharan Africa, the slow pace of progress in fighting hunger over the years is particularly worrisome [3]. This region still holds the highest prevalence of undernourishment for any region, having the number of undernourished people even increased by 44 million between 1990 and 92 and 2014–16 [2].

Some factors associated with malnutrition have been identified in the literature. In the global context, food security, mother and child care (fertility rate and maternal literacy), characteristics of the health services and environment, and potential resources (national and domestic income) were factors explaining the variability in the prevalence of malnutrition among children under 5 years of age in developing countries [4].

Angola, located on the south Atlantic coast of West Africa, is one of the largest and richest countries in the sub Saharan Africa. Its total population, in the census carried out in 2014, was around 25 million inhabitants [5]. After a long civil war, which ended in 2002, the country’s health system is still being rebuilt. In this context, health data of the Angolan population, often obtained through estimates made by nongovernmental organizations operating with the government and health services, are scarce, and scant primary data exist in terms of the determinants of malnutrition. Knowledge of this information is important for proper policy planning to address this problem in a context-specific manner. Thus, the present study aims to describe the nutritional status of children under 2 years of age in a suburban area of Angola, and to identify the factors associated with the occurrence of malnutrition in this population.

## Methods

We carried out a cross-sectional population-based study, linked to a larger project entitled “Developing primary health care services in Angola: a proposal for evaluation of the Community Health Workers Program”, whose data were collected from August 1 to September 26 of 2010 [6]. The study site, Cacuaco, located in the suburban region of Luanda, was chosen because it was the first municipality to implement the Angolan Community Health Workers Program. The estimated population in Cacuaco is 700,000 inhabitants, distributed over an area of 572 km^2^ (population density of 1.2 inhabitants per km^2^).

Participants were recruited in four neighborhoods, which were selected based on the criteria: neighborhood map availability, authorization by resident committees and researchers’ security. The neighborhoods were divided into micro areas, each with 100 households. One house in each micro area was randomly selected as the starting point, and every third house to the right of the index house was visited by the interviewers.

Children under 2 years of age and their mothers were eligible. The exclusion criteria were: mothers who lived for less than 1 year at the study site or who did not live with the child. In the case of more than one child under 2 years of age in the same household, only the oldest was included, since in the original study there was an intention to take advantage of the children’s exposure time to the public health interventions that were being implemented in the area. In case of twins, the child selected was the one born first. In the original study, a sample of 700 children was calculated as necessary, considering the prevalence estimation of main endpoints studied (e.g. children’s low body mass index-for-age and low height-for-age). With this sample size and considering point estimators varying from 10 to 40 percentage points, 95% confidence intervals of < 2.5 percentage points on either side would be attained, considering a conglomerate effect of 1.5. To attain relative risks of the size seen in previous investigations of determinants in other African countries and in Bangladesh [7, 8] for the outcome “malnutrition”, assuming a 5% α-error, power of 80% and the same conglomerate effect, a sample size ranging from 348 to 574 would be necessary.

The Angolan interviewers underwent 5 days of intensive training, after which four teams were assembled, each consisting of a field coordinator, four interviewers, and an area supervisor. A structured questionnaire was applied to the mother and additional data were obtained from pregnancy and child health cards (an English version of the questionnaire is provided in Additional file 1). Standardized anthropometric measurements were obtained by properly trained field coordinators with Tanita® digital scales and custom-made wooden stadiometers.

The outcomes investigated were stunting (low stature for age) and underweight (low weight for age), using the WHO definition of two or more Z scores below the median [9]. Exposure variables surveyed through the questionnaires included: sociodemographic characteristics, economic conditions, living conditions, health situation of the mother, the child and other children in the household, and the use of health services by the mother and the child.

The economic condition was assessed indirectly by means of a score, based on a previous study conducted in Ghana [10], to stratify the participating families into categories of a more or less favored economic situation. Scores were given for certain household characteristics (house building material, piped water, electric light, presence of a refrigerator and of a toilet inside the house) totaling values from 0 to 10.

The questionnaires were coded, scanned and entered into the database using the Teleform® software. In the data analysis, descriptive statistics were initially performed, followed by multivariable analyses using a hierarchical model based on the existing literature [11]. In this model, the exposure variables were classified into levels (distal, intermediate and proximal) considering their proximity to the dependent variable, according to the conceptual basis for possible interrelationships involving the factors under study [12]. As outcomes had high prevalence (greater than 10%), Poisson regression was employed [13] in order to have a better estimate of prevalence ratios and their respective confidence intervals. Moreover, we chose the robust variance model, also known as modified Poisson regression, because it can give a better estimate of the variability of the coefficient estimator when applied to binary outcome variables [14]. At each level of the hierarchical model, the variables were adjusted in relation to the others at the same level. Those with a *p*-value < 0.20 were carried forward to adjust the analyses in the next level. The p-value for statistical significance was 0.05. Statistical analyses were performed with the Statistical Package for the Social Sciences (SPSS) software version 18.0. Missing data were not considered in the analyses, resulting in successive losses in the sample across the levels of the multivariable model.

The study was approved by the Ethics Committee of the Federal University of Rio Grande do Sul (register number 2008045) and by the Provincial Health Department of Luanda, Angola. The interviews were preceded by the signing of an informed consent form by the mother.

## Results

We visited 1360 houses in 49 micro areas of the four selected neighborhoods. Forty-two (5.7%) children whose mothers had lived for less than 1 year at the study site or who did not live with the child were excluded, 111 (15.0%) were lost after three consecutive visits at different days and times, and 10 (1.4%) mothers refused to participate. The final sample included 749 children and their mothers. Table 1 shows the sociodemographic characteristics, as well as the housing conditions and some health features of the study population, together with their crude association with the studied outcomes.

**Table 1:** Sociodemographic and health characteristics of mothers and children under 2 years of age living in the municipality of Cacuaco, Luanda, Angola, and their crude association with underweight and stunting, 2010 (*N* = 749)

Sample characteristics	n^a^ (%) or median	CI 95%^b^ or Q1 - Q3^c^	Crude PR^d^ (95%CI) for underweight^e^	Crude PR (95% CI) for stunting^e^
Mother’s age (years)	25	(21–30)	1.01 (0.99–1.04)	1.01 (0.99–1.03)
Mother’s place of birth				
Luanda Province	76 (10.2)	(12.5–8.1)	1.00	1.00
Another Province	669 (89.8)	(91.4–86.8)	1.04 (0.59–1.85)	1.06 (0.74–1.52)
Marital status				
Not living with partner	110 (14.7)	(17.4–12.2)	1.00	1.00
Living with partner	639 (85.3)	(87.8–82.6)	1.13 (0.67–1.90)	1.45 (1.01–2.10)
Mother’s education level (years of school)				
5 years or more	432 (65.8)	(69.2–62.3)	1.00	1.00
1 to 4 years	225 (34.2)	(37.7–30.8)	0.93 (0.62–1.40)	1.23 (0.98–1.55)
Mother’s occupation				
Self-employed	327 (43.9)	(47.2–40.1)	1.00	1.00
Housewife	316 (42.4)	(45.8–38.6)	1.22 (0.85–1.76)	0.85 (0.68–1.06)
Other	102 (13.7)	(16.2–11.2)	0.61 (0.31–1.20)	0.60 (0.40–0.89)
Partner’s education level (years of school)				
5 years or more	461 (93.1)	(94.8–91.0)	1.00	1.00
1 to 4 years	34 (6.9)	(7.2–6.5)	1.17 (0.45–3.00)	0.72 (0.47–1.10)
Partner’s occupation				
Public sector employee	165 (25.2)	(28.5–22.2)	1.00	1.00
Private sector employee	331 (50.5)	(54.1–46.8)	1.18 (0.76–1.84)	0.90 (0.70–1.16)
Self-employed	129 (19.7)	(22.8–17.0)	0.52 (0.26–1.05)	0.76 (0.54–1.07)
Other	30 (4.6)	(5.0–4.2)	1.09 (0.45–2.65)	0.55 (0.26–1.16)
Gestational age at the onset of prenatal care (in months)	3	(2–4)	1.07 (0.96–1.21)	1.08 (1.01–1.15)
Number of prenatal visits	4	(4–6)	0.99 (0.89–1.09)	0.96 (0.90–1.02)
Place of delivery				
At home	228 (30.5)	(33.9–27.2)	1.00	1.00
At the health service	520 (69.5)	(72.8–66.1)	1.82 (0.57–1.17)	0.79 (0.63–0.98)
Occurrence of death of other children				
No	533 (71.2)	(74.4–67.8)	1.00	1.00
Yes	216 (28.8)	(32.2–25.6)	1.30 (0.91–1.87)	1.20 (0.96–1.50)
Child’s age (in months)	10.12	(4.2–16.0)	1.00 (0.98–1.03)	1.02 (1.01–1.04)
Child’s sex				
Male	361 (49.2)	(52.9–45.6)	1.00	1.00
Female	373 (50.8)	(54.4–47.1)	1.24 (0.87–1.76)	1.07 (0.87–1.33)
Birth weight (in grams)				
< 2500	85 (13.3)	(16.0–11.0)	1.00	1.00
≥ 2500	553 (86.7)	(89.0–84.0)	0.85 (.050–1.43)	0.86 (0.62–1.18)
Exclusive breastfeeding under 6 months				
No	128 (47.6)	(51.3–44.0)	1.00	1.00
Yes	141 (52.4)	(56.0–48.7)	0.89 (0.51–1.54)	0.86 (0.59–1.26)
Breastfeeding under 24 months				
No	110 (14.7)	(17.4–12.2)	1.00	1.00
Yes	638 (85.3)	(87.8–82.6)	0.73 (0.47–1.12)	0.80 (0.61–1.04)
Up to date weight monitoring of the child				
No	292 (39.6)	(43.3–36.1)	1.00	1.00
Yes	446 (60.4)	(63.9–56.7)	0.92 (0.65–1.31)	0.75 (0.60–0.92)
Child with diarrhea in the last 15 days				
No	487 (65.0)	(68.4–61.5)	1.00	1.00
Yes	262 (35.0)	(38.5–31.6)	1.15 (0.81–1.64)	1.37 (1.11–1.69)
Child with fever in the last 15 days				
No	502 (67.0)	(70.4–63.5)	1.00	1.00
Yes	247 (33.0)	(36.5–29.6)	0.98 (0.68–1.43)	1.13 (0.91–1.41)
Presence of mosquito nets (seen by the research staff)				
No	366 (48.9)	(52.5–45-2)	1.00	1.00
Yes	383 (51.1)	(54.8–47.5)	0.90 (0.64–1.27)	1.99 (0.80–1.22)
Child’s main caregiver				
Mother	573 (76.6)	(79.6–73.4)	1.00	1.00
Another person	175 (23.4)	(26.6–20.4)	1.25 (0.85–1.83)	1.29 (1.02–1.62)
Number of people in the household	6	(4–8)	0.96 (0.90–1.04)	1.00 (0.97–1.04)
Presence of hypochloryte in the home (for water treatment)				
No	441 (59.0)	(62.6–55.5)	1.00	1.00
Yes	307 (41.0)	(44.6–37.4)	1.14 (0.80–1.62)	1.01 (0.82–1.25)
Garbage disposal				
Collected by the city	248 (33.2)	(36.7–29.9)	1.00	1.00
Not collected	498 (66.8)	(70.1–63.3)	0.98 (0.68–1.42)	1.06 (0.84–1.33)
Family socioeconomic score	7	(5–7)	0.92 (0.83–1.01)	0.99 (0.93–1.05)

^a^Number of subjects

^b^95% CI: 95% Confidence Interval

^c^Q1-Q3: Intequartile range

^d^ PR: Prevalence Ratio

^e^Underweight and stunting have been defined as binary variables (yes or no) according to the WHO definition of two or more Z scores below the median

Table 2 shows the nutritional status of children, according to anthropometric data measured on the day of the visit, indicating the Z scores for length-for-age and weight-for-age. Considering a Z score below − 2, 232 (32%; 95% CI: 28.7–35.5%) were stunted and 109 (15.1%; 95% CI: 12.6–17.9) were underweight. In Table 2, it is also possible to observe the proportions of severely malnourished children, considering a Z score below − 3.

**Table 2:** Stunting and underweight rates among children under 2 years of age living in the municipality of Cacuaco, Luanda, Angola, 2010 (N = 749)

Nutritional indicators	n^a^ (%)	95% CI^b^
Stunting		
< −3.0 Z scores (very low)	96 (13.2)	10.9–15.9
Between −3.0 and − 2.01 Z scores (low)	136 (18.8)	16.1–21.7
Between −2.0 and − 1.01 Z scores (between low and adequate)	194 (26.8)	23.7–30.2
Between − 1.0 and 1.0 Z scores (adequate)	243 (33.5)	30.1–37.0
Between 1.01 and 2.0 Z scores (between adequate and high)	29 (4.0)	3.6–4.3
Between 2.01 and 3.0 Z scores (high)	13 (1.8)	1.5–2.1
> 3.0 Z scores (very high)	14 (1.9)	1.6–2.2
Underweight		
< −3.0 Z scores (very low)	34 (4.7)	4.3–5.0
Between −3.0 and − 2.01 Z scores (low)	75 (10.4)	8.4–12.8
Between −2.0 and − 1.01 Z scores (between low and adequate)	169 (23.4)	20.4–26.6
Between − 1.0 and 1.0 Z scores (adequate)	375 (52.0)	48.3–55.6
Between 1.01 and 2.0 Z scores (between adequate and high)	48 (6.7)	6.3–7.0
Between 2.01 and 3.0 Z scores (high)	13 (1.8)	1.5–2.1
> 3.0 Z scores (very high)	7 (1.0)	0.8–1.2

^a^Number of subjects

^b^95% CI: 95% Confidence Interval

Tables 3 and 4 show the prevalence ratios (PR) for the studied outcomes, adjusted for the predictors, according to the hierarchical model. For underweight (Table 3), greater gestational age at the onset of prenatal care was the only factor in distal or intermediate models presenting an association. At the proximal level, only the occurrence of death of other children in the household was associated with the outcome. A non-maternal primary caregiver, female sex of the child, and diarrhea during the last 15 days all presented non-statistically significant PRs greater than 1.3. For stunting (Table 4), in distal and intermediate models, mother’s current occupation, with a global *p*-value of 0.109, followed to the next level. In the intermediate model, a mother’s working but not being self-employed was associated with a prevalence 45% lower while the primary caregiver not being the mother was associated with a prevalence 42% higher. In the proximal model, only the occurrence of diarrhea in the last 15 days was associated with the outcome.

**Table 3:** Adjusted associations of risk factors with a child being underweight at different levels of a hierarchical model (the number of missing values has been subtracted from the total N of 749)

Variables	Distal Model PR^a^ (CI 95%^b^) *N* = 629	*p*-value	Intermediate Model PR (CI 95%) *N* = 647	*p*-value	Proximal Model PR (CI 95%) *N* = 549	*p*-value
Mother’s place of birth						
Luanda Province	1.00					
Another Province	1.04 (0.57–1.92)	0.892	–	–	–	–
Mother’s education level (years of school)						
5 years or more	1.00					
1 to 4 years	0.81 (0.53–1.24)	0.331	–	–	–	–
Mother’s occupation						
Self-employed	1.00					
Housewife	1.12 (0.75–1.68)	0.571	–	–	–	–
Other	0.62 (0.31–1.24)	0.176	–	–	–	–
Family socioeconomic score	0.92 (0.82–1.03)	0.155	0.92 (0.83–1.02)	0.113	0.97 (0.86–1.08)	0.559
Mother’s age (years)	–		1.01 (0.98–1.04)	0.607		
Garbage disposal		–			–	–
Collected by the city			1.00			
Not collected	–		0.90 (0.62–1.32)	0.602		
Presence of mosquito nets (as observed)						
No	–	–	1.00			
Yes		–	0.94 (0.65–1.36)	0.731		
Presence of hypochloryte						
No	–	–	1.00		1.00	
Yes	–	–	1.30 (0.90–1.88)	0.162	1.21 (0.80–1.82)	0.369
Child’s main caregiver						
Mother	–	–	1.00		1.00	
Another person			1.35 (0.89–2.03)	0.156	1.49 (0.95–2.35)	0.085
Number of people in the household	–	–	0.93 (0.85–1.01)	0.088	0.94 (0.85–1.03)	0.179
Occurrence of death of other children	–	–				
No	–	–	1.00		1.00	
Yes			1.45 (0.97–2.15)	0.070	**1.52 (1.01–2.29)**	**0.045**
Gestational age at the onset of prenatal care (in months)	–	–	**1.20 (1.03–1.40)**	**0.021**	1.17 (0.98–1.39)	0.084
Number of prenatal visits	–	–	1.13 (0.99–1.29)	0.081	1.10 (0.94–1.28)	0.242
Place of delivery						
At home	–	–	1.00		–	–
At the health service			0.88 (0.60–1.29)	0.510		
Child’s age (in months)	–	–			0.98 (0.95–1.02)	0.312
Child’s sex						
Male	–	–		–	1.00	
Female	–	–	–		1.40 (0.94–2.09)	0.096
Birth weight						
< 2500 g	–	–	–	–	1.00	
> =2500 g			–		1.02 (0.57–1.81)	0.956
Breastfeeding under 24 months						
No	–	–		–	1.00	
Yes			–		0.73 (0.42–1.30)	0.270
Up to date weigth monitoring of the child						
No	–	–		–	1.00	
Yes			–		1.07 (0.68–1.67)	0.774
Child with diarrhea in the last 15 days						
No	–	–	–	–	1.00	
Yes					1.32 (0.85–2.07)	0.220
Child with fever in the last 15 days						
No	–	–	–	–	1.00	
Yes					0.97 (0.61–1.53)	0.890

^a^*PR* Prevalence Ratio, ^b^ *CI 95%* Confidence Interval 95%

The entries in boldface are those with statistical significance (*p* value < 0.05)

**Table 4:** Adjusted associations of risk factors with a child being stunted at different levels of a hierarchical model (the number of missing values has been subtracted from the total N of 749)

Variables	Distal Model PR (CI 95%^b^) *N* = 631	*p*-value	Intermediate Model PR (CI 95%) *N* = 649	*p*-value	Proximal Model PR (CI 95%) *N* = 585	*p*-value
Mother’s place of birth						
Luanda Province	1.00					
Another Province	0.99 (0.68–1.44)	0.946	–	–	–	–
Mother’s education level (years of school)						
5 years or more	1.00					
1 to 4 years	1.11 (0.87–1.41)	0.421	–	–	–	–
Mother’s occupation						
Self-employed	1.00		1.00		1.00	
Housewife	0.90 (0.70–1.14)	0.379	0.94 (0.72–1.21)	0.608	0.88 (0.68–1.14)	0.325
Other	**0.63 (0.41–0.97)**	**0.037**	**0.55 (0.34–0.89**)	**0.014**	0.67 (0.44–1.04)	0.076
Family socioeconomic score	0.97 (0.91–1.04)	0.416	–	–	–	–
Mother’s age (years)	–	–	1.01 (0.99–1.03)	0.432	–	–
Garbage disposal						
Collected by the city			1.00			
Not collected	–	–	0.92 (0.72–1.19)	0.529	–	–
Presence of mosquito nets (as observed)	–	–				
No	–	–	1.00			
Yes	–	–	1.00 (0.79–1.25)	0.981	–	–
Presence of hypochloryte						
No	–	–	1.00		–	–
Yes			1.11 (0.88–1.39)	0.384		
Child’s main caregiver						
Mother	–	–	1.00		1.00	
Another person	–	–	**1.42 (1.10–1.84)**	**0.007**	1.19 (0.90–1.58)	0.228
Number of people in the household	–	–	0.98 (0.94–1.03)	0.393	–	–
Occurrence of death of other children						
No	–	–	1.00			
Yes	–	–	1.14 (0.88–1.47)	0.313	–	–
Gestational age at the onset of prenatal care (in months)	–	–	1.08 (0.96–1.20)	0.199	1.07 (0.99–1.16)	0.099
Number of prenatal visits	–	–	1.03 (0.93–1.13)	0.602	–	–
Place of delivery						
At home	–	–	1.00		1.00	
At the health service	–		0.81 (0.63–1.04)	0.093	0.84 (0.64–1.12)	0.216
Child’s age (in months)					1.00 (0.98–1.03)	0.742
Child’s sex						
Male	–	–	–	–	1.00	
Female	–		–	–	1.16 (0.91–1.47)	0.233
Birth weight						
< 2500 g	–	–	–	–	1.00	
> =2500 g	–		–	–	0.80 (0.58–1.10)	0.167
Breastfeeding under 24 months						
No	–		–	–	1.00	
Yes					0.84 (0.60–1.18)	0.317
Up to date weigth monitoring of the child						
No	–	–	–	–	1.00	
Yes	–	–	–	–	1.01 (1.76–1.34)	0.951
Child with diarrhea in the last 15 days						
No	–		–	–	1.00	
Yes					**1.39 (1.07–1.87**)	**0.015**
Child with fever in the last 15 days						
No	–	–	–	–	1.00	
Yes	–	–	–	–	1.07 (0.81–1.41)	0.619

^a^*PR* Prevalence Ratio, ^b^*CI 95%* Confidence Interval 95%

The entries in boldface are those with statistical significance (*p* value < 0.05)

## Discussion

In our study, the prevalence rates of stunting and underweight in children under 2 years of age was 32 and 15.1%, respectively. Regarding malnutrition’s associated factors, we found, after adjustment for predictors, the occurrence of death of other children and greater gestational age at the onset of prenatal care as risk factors for underweight, and the presence of diarrhea in the last two weeks, as well as mother’s working but not being self-employed and primary caregiver not being the mother, as predictors in the case of stunting.

The prevalence of stunting found in this study, considered high according to WHO standards [15], was slightly higher than that found in the United Nations Children’s Fund (UNICEF) survey conducted in Angola in 2009 (29%), whereas the one found for underweight, considered medium by WHO, was slightly lower (16%) [16]. More recent data released by the Joint Malnutrition Estimates in 2016 show estimates of 4.9% (4.6% in urban area) and 37.6% (31.8% in urban area) of underweight and stunting in Angola, respectively [17].

In the African region, existing data show that the continent has been making slow progress in reducing stunting over time. From 2000 to 2015, although stunting prevalence among children under 5 decreased from 38 to 32%, the number of stunted children increased from 50.4 million to 58.5 million [17]. Considering that the first of six global targets set by Member States in the 65th World Health Assembly, to be achieved by 2025, is to reduce by 40% the number of stunted children, then the region is not making a good response [17].

Our findings showed that children of mothers with a history of at least one death among the previous children had a higher prevalence of underweight. This variable seems to reflect the number of people living in the house, since mothers with deceased children had a higher average number of people living in the household (6.99 vs. 6.22, *p* < 0.001; univariate analysis, data not shown). In this case, our finding is in line with other studies in different countries that found that children of mothers with more children (indirectly reflecting more people living in the household) have more malnutrition [7, 18–20]. We found that early onset of prenatal care protected children from being underweight, like other studies, in Ghana and in Brazil, that showed that improved access to prenatal care was associated with a lower prevalence of malnutrition [21, 22].

We also observed the association of diarrhea in the last two weeks with stunting, but not with underweight, partially corroborating with studies in Ethiopia [7] and Bangladesh [8]. The mother’s occupation (another in relation to self-employed) obtained statistical significance in the intermediate model. In any case, mothers with other occupations (with formal employment or students), who are not self-employed nor housewives, usually have more resources, both financial and related to organization of daily life, which could influence the nutritional status of their children. Hien and Hoa found an independent association of maternal occupation (peasant mothers) with a higher risk of malnutrition [18]. In our analysis, the main caregiver (the mother compared to another) presented statistical significance in the intermediate model (*p* = 0.007), suggesting that the presence of the mother taking care of the child has a protective role in relation to malnutrition.

We identified two risk factors not mentioned in other studies – mother working in a non self-employed position and non maternal caretaker. This is an original contribution of our study, which probably makes more sense in the modern life style and in the urban setting, and it highlights the need to discuss about the best strategies to help mothers to better structure and organize their lives in the postpartum period, such as having a protected maternal leave followed by formal job opportunities.

Unlike other studies [18, 20, 22, 23] we have not identified low birth weight as a determinant of malnutrition, possibly because the data collected was self-reported, therefore with reduced reliability. We also did not find an association of maternal schooling nor of economic situation with the evaluated outcomes, like other authors did [7, 11, 19–22]. It is likely that our population is very homogeneous in economic terms, and that the difference in years of school, in the context studied, does not represent a real difference in the life of families, given the low quality of education in general. We did not find any association with the child’s sex, contrasting with two studies that have identified the male sex as a risk factor for malnutrition [7, 8, 24].

Our study has some limitations. It is important to note that, although the study had a sample of 749 mothers and children, in many of the variables, the N was much smaller, and this would impair the multivariable model. Thus, a few variables, such as exclusive breastfeeding below 6 months (*N* = 269), were not included in the model due to the small number of observations. In addition, the occurrence of successive losses in the sample can be observed in the multivariable analysis, due to missing values in one or another variable.

Despite these limitations, this is an unprecedented work whose findings bring relevant contributions to the health policies focused on improving the nutritional status of children in Angola and in other countries with similar contexts. Our results point to the importance of strengthening family planning policies and to the need for improvement in primary health care and sanitation, because of the high prevalence of malnutrition found, especially stunting, which has been associated with the occurrence of diarrhea in the last two weeks. Due to the study’s cross-sectional design, we cannot affirm causality, so it is possible that children with recent diarrhea have had repeated episodes previously, leading to chronic malnutrition and stunting. Or that due to pre-existing nutritional deficits, these children are more vulnerable to infections that lead to diarrhea. According to Rissin et al. (2006), malnutrition can be considered a timely disease of recurrent infectious diseases, and in this view, recurrent diarrhea has been shown to be a potential determinant of malnutrition, due to the decreased nutrient absorption imposed on the organism [25].

It is also worth mentioning that the studied population was collectively exposed to several factors that may influence their nutritional status. Aside from lack of access to quality primary care and education, as mentioned previously, these include endemic malaria, precarious sanitation and lack of food. These exposures, because of their almost universal character in the context of our study, were not measured. Therefore, we do not know how much they are involved with the high prevalence of malnutrition at the study site, but the fact that we identified relatively few individual risk factors speaks in favor of the importance of collective exposures. Nevertheless, it is important to highlight the effort of revitalization of the Municipal Health System in Cacuaco [26], whose deployment began in 2007, including the Community Health Workers Program [6, 27]. The findings concerning this suburban area in Luanda cannot be generalized to the whole country, but they provide a scenario that is probably similar to other suburban areas in Angola, especially those surrounding the capital city. Additionally, these results shall be insightful for other countries as well, especially in Sub-Saharan Africa, where resembling contexts are likely to be found.

We are currently living the post 2015 development era, and estimates of child malnutrition are useful for monitoring progress towards the Sustainable Development Goals, in particular “ending hunger, achieving food security, and improving nutrition and promoting sustainable agriculture” [2]. In Africa, the variation in stunting prevalence was 40.5% (1980) [26] to 32% (2015) [17]. According to prominent researchers, investing in interventions aimed at improving physical growth and mental health of children is important not only to reduce the prevalence of malnutrition but also to avoid its negative functional consequences throughout the life cycle [28] and to be able to build a favorable human capital [29].

Our findings also cause reflection on the nutritional transition, a worldwide trend. It is important to note that food insecurity is complex, leading to recurrent malnutrition and hunger crises, but also to overeating and eating errors, which can lead to overweight and obesity. Therefore, it is necessary to be alert to the double burden of malnutrition caused by the vicious circle of poverty, hunger and food insecurity, facing its determinants and prioritizing the implementation of public policies that can prevent such illnesses, as is being pointed out in this study.

## Conclusions

The high prevalence of stunting and underweight found in this study enable us to conclude that malnutrition is still an important problem among children under 2 years in Angola. The absence of strong individual risk factors in our study suggests that a combination of life course factors, particularly those associated with pregnancy and birth, which we could not accurately measure, and collective exposures, over which individuals have little control, likely play a predominant role. Thus, a joint and coordinated effort between government, community, and nongovernmental organizations operating in the country is necessary to improve the nutritional status of children, focusing on effective programs and policies that reinforce the removal of collective risk factors such as lack of safe water and basic sanitation, and the provision of adequate and accessible education and health services to the population to enable effective health education actions as well as prevention and treatment of child malnutrition at the individual level.

## Additional file

Additional file 1: Study Questionnaire. Structured questionnaire applied to participant mothers in data collection. The questionnaire was applied by trained interviewers. Anthropometric information was collected by field coordinators, who also revised the whole content of the interview and finalized the questionnaires. (PDF 576 kb)

## Abbreviations

BMI: Body mass index
PR: Prevalence ratio
SPSS: Statistical package for the social sciences
UNICEF: United nations children’s fund
WHO: World health organization

## References

[1] World Health Organization. Essential nutrition actions: improving maternal, newborn, infant and young child health and nutrition. Geneva: WHO; 2013 [accessed in 2019 11 Jan]. Available from: http://apps.who.int/iris/bitstream/10665/84409/1/9789241505550_eng.pdf. 25473713

[2] United Nations. Food and Agriculture Organization, International Fund for Agricultural Development, World Food Programme. The state of food insecurity in the world: meeting the 2015 international hunger targets: taking stock of uneven progress. Rome: FAO; 2015 [accessed in 2019 14 Jan]. Available from: http://www.fao.org/3/a-i4646e.pdf.

[3] Requejo JH, Bryce J, Barros AJD, Berman P, Bhutta Z, Chopra M (2015). Countdown to 2015 and beyond: fulfilling the health agenda for women and children. Lancet.

[4] Frongillo EA, de Onis M (1997). Hanson KM (1997) socioeconomic and demographic factors are associated with worldwide patterns of stunting and wasting of children. J Nutr.

[5] ANGOP Angolan Press Agency [Angola: Angolan population reaches 25 million inhabitants, according to definitive census data] News from 2016, 23 Mar [accessed in 2019 18 Jan]. Available from: http://www.angop.ao/angola/pt_pt/noticias/sociedade/2016/2/12/Angola-Populacao-angolana-atinge-milhoes-habitantes-segundo-dados-definitivos-censo,790199af-5cb1-43e2-8583-def58f1873c2.html.

[6] Humbwavali JB, Giugliani C, Duncan BB, Harzheim E, Lavor ACH, Lavor MC (2014). Health and health Care of Mothers and Children in a suburban area of Luanda, Angola. J Community Health.

[7] Asfaw M, Wondaferash M, Taha M, Dube L. Prevalence of undernutrition and associated factors among children aged between six to fifty nine months in Bule Hora district, South Ethiopia. BMC Public Health. 2015;15:–41. 10.1186/s12889-015-1370-9 PMC4314803 25636688

[8] Ahmed AMS, Ahmed T, Roy SK, Alam N, Hossain MI (2012). Determinants of undernutrition in children under 2 years of age from rural Bangladesh. Indian Pediatr.

[9] World Health Organization. WHO Child Growth Standards: Length/height-for-age, weight-for-age, weight-for-length, weight-for-height and body mass index-for-age: Methods and development. Geneva: WHO; 2006 [accessed in 2019 14 Jan]. Available from: https://www.who.int/childgrowth/standards/Technical_report.pdf

[10] Krefis AC, Schwarz NG, Nkrumah B, Acquah S, Loag W, Sarpong N (2010). Principal component analysis of socioeconomic factors and their association with malaria in children from the Ashanti region, Ghana. Malar J.

[11] Aerts D, Drachler ML, Giugliani ERJ (2004). Determinants of growth retardation in southern Brazil. Cad Saúde Pública.

[12] Victora CCG, Huttly SRS, Fuchs SCS, Olinto MMTA (1997). The role of conceptual frameworks in epidemiological analysis: a hierarchical approach. Int J Epidemiol.

[13] Coutinho LMS, Scazufca M, Menezes PR (2008). Métodos para estimar razão de prevalência em estudos de corte transversal. Rev Saúde Pública.

[14] Barros AJD, Hirakata VN (2003). Alternatives for logistic regression in cross-sectional studies: an empirical comparison of models that directly estimate the prevalence ratio. BMC Med Res Methodol.

[15] World Health Organization [internet]. Nutrition Landscape Information System (NLiS). Geneva: Nutrition; 2016 [accessed in 2019 14 Jan]. Available from: http://www.who.int/nutrition/nlis/en/.

[16] United Nations Children’s Fund. The state of the world’s children 2016: a fair chance for every child. New York: UNICEF; 2016 [accessed in 2019 14 Jan]. Available from: https://www.unicef.org/publications/files/UNICEF_SOWC_2016.pdf

[17] United Nations Children’s Fund, World Health Organization, World Bank Joint Child Malnutrition Estimates (JME). [accessed in 2019 11 Jan]. Available from: https://data.worldbank.org/indicator/SH.STA.MALN.ZS?locations=AO

[18] Hien N, Hoa N (2009). Nutritional status and determinants of malnutrition in children under three years of age in Nghean, Vietnam. Pak J Nutr.

[19] Drachler ML, Andersson MCS, Leite JCC, Marshall T, Freitas PF, Aerts DRGC (2003). Social inequality and other determinants of height in children: a multilevel analysis. Cad Saúde Pública..

[20] Vitolo MR, Gama CM, Bortolini GA, Campagnolo PDB, Drachler ML (2008). Some risk factors associated with overweight, stunting and wasting among children under 5 years old. J Pediatr.

[21] Van de Poel E, Hosseinpoor AR, Jehu-Appiah C, Vega J, Speybroeck N (2007). Malnutrition and the disproportional burden on the poor: the case of Ghana. Int J Equity Health.

[22] Oliveira VA, Assis AMO, Pinheiro SMC, Barreto ML (2006). Determinants of weight and linear growth deficits in children under two years of age. Rev Saúde Pública..

[23] Mamiro PS, Kolsteren P, Roberfroid D, Tatala S, Opsomer AS, Van Camp JH (2005). Feeding practices and factors contributing to wasting, stunting, and iron-deficiency anaemia among 3-23-month old children in Kilosa district, rural Tanzania. J Health Popul Nutr.

[24] Rakotomanana H, Gates GE, Hildebrand D, Stoecker BJ (2017). Determinants of stunting in children under 5 years in Madagascar. Matern Child Nutr.

[25] Rissin A. [Malnutrition in children under five in the state of Pernambuco: an analysis of hierarchized causal relations] [dissertation]. Recife, PE: Universidade Federal de Pernambuco; 2003.

[26] Angola (2008). Ministry of Health. [National Direction of Public Health. Revitalization of Municipal Health Services to Accelerate the Reduction of Maternal and Child Mortality]. Luanda: Ministry of Health; 2008.

[27] Giugliani C, Duncan BB, Harzheim E, Lavor ACH, Lavor MC, Machado MMT (2014). Community health workers programme in Luanda, Angola: an evaluation of the implementation process. Hum Resour Health.

[28] Onis M, Frongillo EA (2000). Blössner M. Is malnutrition declining? An analysis of changes in levels of child malnutrition since 1980. Bull World Health Organ.

[29] Victora CG, Adair L, Fall C, Hallal PC, Martorell R, Richter L (2008). Maternal and child undernutrition: consequences for adult health and human capital. Lancet.

