# Supplementary material for: Malnutrition and its associated factors: a cross-sectional study with children under 2 years in a suburban area in Angola
Source: BMC Public Health. 2019 Feb 21;19:220. doi: 10.1186/s12889-019-6543-5 (PMC6385448; doi:10.1186/s12889-019-6543-5)
Supplement: Supplementary file 1 — Study Questionnaire. Structured questionnaire applied to participant mothers in data collection. The questionnaire was applied by trained interviewers. Anthropometric information was collected by field coordinators, who also revised the whole content of the interview and finalized the questionnaires. (PDF 576 kb) [file 12889_2019_6543_MOESM1_ESM.pdf]

Identification #

THIS IS AN ENGLISH VERSION OF THE QUESTIONNAIRE MADE UNDER REQUEST AND IT HAS NOT BEEN FORMATTED FOR THE TELEFORM SOFTWARE

FEDERAL UNIVERSITY OF DO RIO GRANDE DO SUL
FACULTY OF MEDICINE
GRADUATE PROGRAM IN EPIDEMIOLOGY

Evaluation of the Impact of Luanda's Community Health Workers Program in the Municipality of Cacuaco

INDIVIDUAL QUESTIONNAIRE

COORDINATOR ADMINISTRATIVE INFORMATION

Coordinator Code __

Microarea Code __ __ __
In the first digit, write down: 0 for area "without CHW"
1 for area "with CHW"

Microarea name: ____

Adress: ____

Telephone: ____________________

INTERVIEWER ADMINISTRATIVE INFORMATION

Interviewer Code: __ __

Interview Date: ____/___/_______

INTRODUCTION

When you arrive at the mother's house, introduce yourself and tell the reason for the visit: *I am part of a research team that is assessing the health situation in Cacuaco and would like to talk to you.*

Then ask: *Have you already received any visitors here informing about this conversation?*

Explain again the reason for the interview: *The purpose of this interview is to talk directly with the mothers of young children in this community about their health situation. These conversations will help us to know what is good and what needs to be improved in the health care of the population, especially children and pregnant women.*

Next, explain the Consent Form: *Before we start with the questions, let's read a letter to make sure there are no doubts about this interview. A copy of this letter will remain with you and another copy will remain with the research team.*

Read the Consent Form, collect the interviewee's signature on the two copies and do not forget to identify it with the same number given to the questionnaire in its upper right corner (Identification).

Then, proceed in applying the questionnaire:

DATA FROM THE MOTHER

1. What is your full name? ____

2. How old are you? _ _

3. Where were you born ?
   In this same Municipality ( )
   In another Municipality in the Province of Luanda ( ) Which one:_________________
   In another Province ( ) Which one: ____________
   In another Country ( ) Which one: ___________

4. How long have you been living in this neighborhood?
   More than 2 years ( )
   Between 1 and 2 years ( )
   Less than 1 year ( )

5. What is your profession?____________

6. What is your current occupation
   Housewife ( )
   Works in the public function ( )
   Works in the private sector ( )
   Autonomous (sales, general services) ( )
   Student ( )
   Other occupation ( ) Which one:______________________

7. Do you know how to read and write? Yes ( ) No ( ) Just my name ( )

8. Have you gone to school? Yes ( ) No ( )
   If YES, until what level did you go to (and concluded)?: __ __

9. Are you: Married/Living with your partner ( ) Separated/divorced ( ) Single ( ) Widow ( )

GENERAL DATA ABOUT THE RESIDENTS OF THE HOUSE

10. How many people live in the house with you? _____ people
    Husband/Partner: Yes ( ) No ( )
    Children: Yes ( ) No ( ) How many: ______
    Grandparents of the child: Yes ( ) No ( ) How many: ______
    Other relatives: Yes ( ) No ( ) How many: ______
    Other (not relatives): Yes ( ) No ( ) How many: ______

If doesn't have husband/partner, go to question 14

11. Has your husband/partner gone to school? Yes ( ) No ( ) Don't know ( )
    If YES, until what level did he go to (and concluded): __ __ Don't know ( )

12. What is the profession of your husband/partner? ____________

13. What is the current occupation of your husband/partner?
Works in the public function ( )
Works in the private sector ( )
Autonomous (sales, general services) ( )
Student ( )
Unemployed ( )
Retired ( )
Other occupation ( ) Which one:________________________________

14. What is the monthly income of your household? (including all those who contribute with some income in the house)

Person 1		Kwanzas/ month	Person 7		Kwanzas/ month
Person 2		Kwanzas/ month	Person 8		Kwanzas/ month
Person 3		Kwanzas/ month	Person 9		Kwanzas/ month
Person 4		Kwanzas/ month	Person 10		Kwanzas/ month
Person 5		Kwanzas/ month	Person 11		Kwanzas/ month
Person 6		Kwanzas/ month	Person 12		Kwanzas/ month

COORDINATOR: Calculate total monthly household income: _________ kwanzas

DATA ABOUT THE MOTHER AND THE CHILDREN

15. How many children did you have? ____ children (consider all children, including those who have passed away)
16. Write down the names of all children, from the oldest to the youngest, including the selected child, and complete the information (in the case of twins, put the one that was born first on front):

Order	Name	Age in **months**	Age in **years**	Day, month and year of birth	Current condition	Lives with you?
Child 1				__/__/_____<br>Don't know ( )	Alive ( ) Deceased ( )<br>Stillborn ( )	Yes ( )<br>No ( )
Child 2				__/__/_____<br>Don't know ( )	Alive ( ) Deceased ( )<br>Stillborn ( )	Yes ( )<br>No ( )
Child 3				__/__/_____<br>Don't know ( )	Alive ( ) Deceased ( )<br>Stillborn ( )	Yes ( )<br>No ( )
Child 4				__/__/_____<br>Don't know ( )	Alive ( ) Deceased ( )<br>Stillborn ( )	Yes ( )<br>No ( )
Child 5				__/__/_____<br>Don't know ( )	Alive ( ) Deceased ( )<br>Stillborn ( )	Yes ( )<br>No ( )
Child 6				__/__/_____<br>Don't know ( )	Alive ( ) Deceased ( )<br>Stillborn ( )	Yes ( )<br>No ( )
Child 7				__/__/_____<br>Don't know ( )	Alive ( ) Deceased ( )<br>Stillborn ( )	Yes ( )<br>No ( )
Child 8				__/__/_____<br>Don't know ( )	Alive ( ) Deceased ( )<br>Stillborn ( )	Yes ( )<br>No ( )
Child 9				__/__/_____<br>Don't know ( )	Alive ( ) Deceased ( )<br>Stillborn ( )	Yes ( )<br>No ( )
Child 10				__/__/_____<br>Don't know ( )	Alive ( ) Deceased ( )<br>Stillborn ( )	Yes ( )<br>No ( )
Child 11				__/__/_____	Alive ( ) Deceased ( )	Yes ( )

				Don't know ( )	Stillborn ( )	No ( )
Child 12				__/__/_____<br>Don't know ( )	Alive ( ) Deceased ( )<br>Stillborn ( )	Yes ( )<br>No ( )

17. Have you ever had an abortion (before completing 5 months of pregnancy)?

    Yes ( ) How many: ___ No ( ) Don´t know ( ) Don't remember ( )

18. Are you pregnant now? Yes ( ) No ( ) Don't know ( )

19. If you are pregnant, how many months of pregnancy do you estimate? ____ months
    Don't know ( ) Not pregnant ( )

COORDINATOR: Calculate the age range between the selected child and his / her younger brother / sister: _____ months Doesn't have younger brother / sister: ( )

COORDINATOR: Calculate the age range between the selected child and his / her older brother / sister:_____ months Doesn't have older brother / sister: ( )

COORDINATOR:

Nº of alive children	
Nº of deceased children	
Nº of stillborns	
Nº of children living with the mother	
Nº of abortions	
TOTAL number of pregnancies (not counting the current pregnancy)	

DATA ABOUT PREGNANCY AND CHILDBIRTH OF THE SELECTED CHILD

Ask the mother directly (do not look at the child's card at this time):

20. What is the full name of the child? ____

All the questions I'm going to ask now are about (child's name) pregnancy.

21. Did any midwife or birth attendant follow your pregnancy at home?
    Yes ( ) No ( )

22. Did you attend antenatal visits when you were pregnant?
    Yes, in the health unit or neighborhood health center ( )
    Yes, in the public hospital ( )
    Yes, in the private clinic or hospital ( )
    Yes, in another place ( ) which one:_______________
    No ( )

23. If you did not have prenatal care, what was the reason?
    Did not have time to go ( ) Did not have access to a visit ( ) Too far ( ) Did not have money ( )
    Did not need, as did not have any problem in pregnancy ( )
    Another reason ( ) Which one: _________

If did not have prenatal care, skip to question 28.

24. With how many months of pregnancy did you start your prenatal care? ___ months

25. How many prenatal visits did you have? ___ visits
Don’t remember ( )

26. Were you weighed in all prenatal visits? **(Read all the alternatives)**
Yes, in all visits ( ) Yes, but not in all visits ( )
Yes, in only one visit ( ) No ( ) Don’t remember ( )

27. Have you had your blood pressure measured at prenatal visits?? **(Read all the alternatives)**
Yes, in all visits ( ) Yes, but not in all visits ( )
Yes, in only one visit ( ) No ( ) Don’t remember ( )

28. Was your pregnancy twin? Yes ( ) No ( )

29. Did you get the tetanus vaccine during pregnancy? **(Read all the alternatives)**
Yes, 1 dose ( ) Yes, 2 doses ( ) Yes, 3 doses ( ) I did not need it, because I was already vaccinated ( ) No ( )
Don’t remember ( )

30. Have you taken medication or tablets for deworming (albendazole) during pregnancy? Yes ( )
No ( ) Don’t know ( ) Don’t remember ( )

31. Have you taken a malaria drug (Fansidar or other) in pregnancy?
Yes, 2 or more doses ( ) Yes, 1 dose No ( ) Don’t know ( ) Don’t remember ( )

32. Have you taken medicine for anemia (iron salt and / or folic acid) during pregnancy? Yes ( ) No ( )
Don’t know ( ) Don’t remember ( )

33. Did you get sick during pregnancy? Yes ( ) No ( ) Don’t remember ( )
If YES, what problem did you have? **(Read all the alternatives)**
Malaria ( ) Hemorrhage ( ) High blood pressure ( ) Pneumonia/tuberculosis ( ) Diarrhea / intestinal infection ( ) Other ( ) Which one:____________________________________

34. Were you hospitalized during pregnancy? Yes ( ) No ( ) Don’t know ( )
If YES, for how many days:___

35. Have you been transferred from the health unit to the hospital or maternity hospital?
Yes ( ) No ( ) Don’t know ( ) Don’t remember ( )
If YES, what was the reason? ____

36. Where was the birth of (child's name)? In the neighborhood health unit ( ) In the hospital ( )
At home ( ) In another place ( ) Which one: _______

37. How long were you in the health unit or hospital after delivery?___ hours
Don’t remember ( ) not applicable ( )

38. The delivery was: vaginal ( ) with forceps ( ) with suction cup / vacuum ( ) caesarean ( )
Don’t know ( )
If cesarean, specify the reason:____________________________ Don’t know ( )

39. The time of delivery was: 9 months ( ) preterm ( ) more than 9 months ( ) Don’t know ( )

40. Did you have any problems at childbirth? Yes ( ) No ( ) Don't know ( )
If YES, what problem did you have? **(Read all the alternatives)**
Hemorrhage ( ) High blood pressure ( ) Seizure ( ) Malaria ( ) High fever/infection ( )
Other ( ) Which one:____________ Don't know ( )

41. Did you have an appointment at the health unit or hospital within the first week after delivery?
Yes ( ) No ( ) Don't remember ( )

42. How long after the birth did you attend to a visit?___ days Don't remember ( ) not applicable ( )

43. Did you get sick within 10 days of giving birth? Yes ( ) No ( ) Don't remember ( )
If YES, what problem did you have? **(Read all the alternatives)**
Hemorrhage ( ) High blood pressure ( ) Seizure ( ) Malaria ( ) High fever/infection ( )
Pneumonia/tuberculosis ( ) Other ( ) Which one:____________ Don't know ( )

44. Were you hospitalized due to complications at delivery or soon after delivery? Yes ( ) No ( )
Don't remember ( )

45. Did you pay for any prenatal visits or to do blood tests and take prenatal medications?
Yes ( ) No ( ) Don't remember ( )

46. Did you pay to deliver?
Yes ( ) No ( ) Don't remember ( )

DATA ABOUT THE REGISTER OF THE SELECTED CHILD

47. (Child's name) has already been registered or has a registration card? Yes ( ) No ( )
Don't know ( )

**If registered child, ask:**	**If child not registered, ask**
48. At what age was she / he registered?<br>In the first week after delivery ( )<br>In the first month of life ( )<br>Before completing 1 year ( )<br>After the first year( )	51. If not registered, what is the reason?<br>Have to pay ( )<br>Distance ( )<br>Did not know that has to register ( )<br>Did not know where to register ( )<br>The wait is long ( )<br>Other reason ( )<br>Which one:___________
49. Where was she/he registered?<br>In the Health Unit or Hospital, before discharge ( )<br>In the Health Unit or Hospital, after discharge ( )<br>In the Health Unit or Hospital, after delivery at home ( )<br>In the Municipal Administration ( )<br>Other ( ) Which one:______________________	
50. Did anyone help you to register the child?<br>Yes ( ) No ( )<br>If YES, who helped: _____________________	

DATA ABOUT THE HEALTH OF THE SELECTED CHILD

Ask the mother directly (do not look at the child's card):

52. Who takes care of (child's name) most of the time?
Mother ( ) Grandmother ( ) Sister/brother ( ) Another relative ( ) Who: ____________
Another person ( ) Who: ___________ Goes to nursery ( )

53. Do you know what the birth weight of (child's name) was? _____ grams Don't know ( )

54. (Name of the child) was hospitalized shortly after birth?
Yes ( ) No ( ) Don't know ( )
If YES, what was the reason? ______________________________
If YES, for how many days? ___

55. (Child's name) has already made the BCG vaccine (mark on the arm) : Yes ( ) No ( )
Don't know ( )
If YES, where? In the Health Unit ( ) Campaign ( ) Other ( ) Which one:______________

56. (Child's name) has already made the Polio vaccine (droplets through the mouth): Yes ( ) No ( )
Don't know ( )
If YES, how many times? 4 or more ( ) 3 ( ) 2 ( ) 1 ( ) Don't know ( )
If YES, where? In the Health Unit ( ) Campaign ( ) Other ( ) Which one:______________
(If more than 1 dose, consider where the last dose was taken)

57. (Child's name) has already made the Pentavalent / DTP vaccine (on the thigh): Yes ( ) No ( )
Don't know ( )
If YES, how many times? 3 or more ( ) 2 ( ) 1 ( ) Don't know ( )
If YES, where? In the Health Unit ( ) Campaign ( ) Other ( ) Which one:______________
(If more than 1 dose, consider where the last dose was taken)

58. (Child's name) has already made the measles vaccine? (on the back): Yes ( ) No ( )
Don't know ( )
If YES, where? In the Health Unit ( ) Campaign ( ) Other ( ) Which:______________

59. (Child's name) has already made the yellow fever vaccine (on the arm): Yes ( ) No ( )
Do not know ( )
If YES, where did? In the Health Unit ( ) Campaign ( ) Other ( ) Which:______________

60. (Child's name) received Vitamin A (reddish droplets from the mouth)? Yes ( ) No ( )
Do not know ( )
If YES, how many times? 2 or more ( ) 1 ( ) Do not know ( )
If YES, where did? In the Health Unit ( ) Campaign ( ) Other ( ) Which:______________
(If more than 1 dose, consider where the last dose was taken)

61. If the child is under 1 year, skip this question and check NA ( )
(Name of child) received Albendazole? Yes ( ) No ( ) Don't know ( )
If YES, how many times? 3 or more ( ) 2 ( ) 1 ( ) Don't know ( )
If YES, where? In the Health Unit ( ) Campaign ( ) Other ( ) Which:______________
(If more than 1 dose, consider where the last dose was taken)

62. (Child's name) has a bednet? **(Read the alternatives)**
Yes ( ) No ( ) Don't know ( )
If YES, how did you get it? Received in the Health Unit ( ) Received from CHW ( ) Purchased ( )
Other ( ) Which one:______________ Don't know ( )

63. When (child's name) was last weighed? In the last month ( ) 1 to 3 months ( ) 4 to 6 months ( )
7 to 12 months ( ) more than 12 months ( ) Not weighed( )

64. Have you breastfed or are you still breastfeeding? Yes ( ) No ( )

65. When was the first time (child's name) suckled?
As soon as she/he was born (in the first hour of life) ( ) In the first 24 hours ( ) After 24 hours ( )
Never breastfed ( )

Ask if the child (ask one by one) uses each of the following items and when she/he started using:

Type of liquid or food	Already used?		When did she/he first use it?		
	YES	NO	DAYS	MONTHS	Don't know
66. Water					
67. Tea					
68. Juice					
69. Soft drink					
70. Kissangua					
71. Other milk					
72. Food (any food: pap, *pirão, funje*, fruit, bean, carrot or other food)					

73. How old was (child's name) when she/he stopped breastfeeding?__ months __ days
Don't know ( ) Still breastfeeds ( )

COORDINATOR: Calculate the time of exclusive breastfeeding:____ days
Calculate the total time of breastfeeding:____ months

74. Has (Name of the child) already attended a medical visit without being ill? Yes ( ) No ( )
Don't know ( )
If YES, where was the visit?
Health Unit or Public Hospital ( ) Health Unit, clinic or Private Hospital ( )
Traditional Center ( ) Other ( ) Which one: _____________
If YES, what was the reason for the visit? ____

75. **Ask if the child had any of the following conditions and needed assistance:**

	(Name of child) had any of these problems IN THE LAST 15 DAYS?		
IF YES, what happened??<br>Mark with an X in the column of each problem	DIARRHEA<br>Yes ( ) No ( )	FEVER<br>Yes ( ) No ( )	COUGH or SHORTNESS OF AIR<br>Yes ( ) No ( )
Was taken to the emergency unit / public hospital			
Was taken to the health unit, clinic or private hospital			
Was taken to traditional center / traditional treatment / church			
Other location (store, pharmacy or other) Which one: _____________			

No need to seek assistance, used home methods			
No need to seek care, used medicines on their own			
There was no need to seek assistance or use any method or medication			

76. In the last 3 months, did you have to bring (name of the child) for a visit at the Health Unit because of illness? Yes ( ) No ( ) Don't remember ( ) If Yes, how many times? _____

77. In the last 3 months, did you need to stay at home taking care of (name of child) due to illness?

    Yes ( ) No ( ) Don't remember ( ) If Yes, how many times? _____

78. (Name of child) ever had to be hospitalized in the last 12 months? Yes ( ) No ( ) Don't remember ( )
    If Yes, how many times? _____

If YES, what was the reason for the hospitalization?

Mark with an X the reason for each hospitalization	Malaria	Diarrhea / dehydration	Respiratory infection	Other _________	Other _________	Don't know
Hospitalization 1						
Hospitalization 2						
Hospitalization 3						
Hospitalization 4						

79. Have you paid for any visits to (name of child) at the Public Health Unit? Yes ( ) No ( ) Don't remember ( )
    If YES, when was it? More than 2 years ago ( ) Between 1 and 2 years ago ( ) Between 1 year and 6 months ago ( ) Less than 6 months ago ( )
    If YES, how much did you pay? __ __ kwanzas

80. Have you ever paid for drugs delivered to (child's name) at the Health Unit? Yes ( ) No ( ) Don't remember ( )
    If YES, when was it? More than 2 years ago ( ) Between 1 and 2 years ago ( ) Between 1 year and 6 months ago ( ) Less than 6 months ago ( )
    If YES, how much did you pay? __ __ kwanzas

81. Have you ever bought medicines to (child's name) outside the Health Unit? Yes ( ) No ( ) Don't remember ( )

82. How have you gotten to the Health Unit? On foot ( ) By bike ( ) Motorcycle ( ) Taxi cab ( ) Private car ( ) other ( ) Which one:_____________________

83. How long does it take you to get to the Health Unit with this means of transportation? _____ minutes

84. Has it ever happened that you had to take (name of the child) to consult with the Health Unit because of illness, but could not? Yes, it happened ( ) No, never happened ( ) I do not remember ( )
    If YES, what was the reason? Distance - had no means to go ( ) Thought she was going to have to pay for the consultation and had no money ( ) Had no one to take care the child ( )

DATA ABOUT THE HEALTH OF THE OTHER CHILDREN

85. Did any other children under the age of five have ever been hospitalized in the last 12 months?
Yes ( ) No ( ) Do not know ( ) If YES, how many children were hospitalized? ___

86. List the children who have been hospitalized in the last 12 months (list more than once if the same child has been hospitalized more than once) and mark the reason for hospitalization with an X:

Name of hospitalized child	Malaria	Diarrhea/ dehydration	Respiratory infection	Other _________	Other _________	Don't know

87. In case of deceased children, list each death and mark with an X the reason for death:

Name of deceased child	How old was she/he when passed away?		How long has he passed away?		Malaria	Diarrhea / dehydration	Respiratory infection	Other ______	Other _______	Don't know
	Months	Years	Months	Years						

88. Did you get sick during pregnancy, delivery or shortly after the birth of one of the other children? Yes ( ) No ( ) Don't know ( )
If YES, in how many of them:____
If YES, what problem did you have?______________

Mark with an X the reason for each pregnancy / delivery	Malaria	Hemorrhage	High Bood Pressure	Seizures	High fever/infection	Other ______	Other ______	Don't know
Pregnancy/delivery 1								
Pregnancy/delivery 2								
Pregnancy/delivery 3								
Pregnancy/delivery 4								
Pregnancy/delivery 5								
Pregnancy/delivery 6								

DATA ABOUT THE HOUSE AND LIVING CONDITIONS

89. Is your house: owned ( ) leased ( ) ceded ( ) taken ( )

90. What is the building material of the house? brick / blocks ( ) Wood ( ) *Adobe* ( ) Other ( )
Which: __________________

91. What is the material on the floor of the house? Natural floor (clay / sand) ( ) Wood / straw / bamboo boards ( ) Ground / finished floor ( ) Other ( ) Which: __________________

92. How many rooms or compartments does the house have? ____

93. Is there a bathroom/toilet? Yes ( ) No ( ) How many: ____

94. The bathroom/toilet is: inside the house ( ) outside the house – exclusive use ( ) outside the house – common use with other houses ( ) There is no bathroom/toilet ( )

95. How is the drainage of the bathroom/toilet?
Sewer system (sink, toilet) ( )
Septic tank with broken well ( )
Dry latrine ( )
There is no bathroom/toilet ( )

96. If you do not have a bathroom/toilet, what do you use in case of need?
Black/ open trench ( )
Direct to the river, lake or sea ( )
Bucket ( )
Grass, bush, open air ( )
There is a bathroom/toilet ( )

97. Does your house have piped water? Yes ( ) No ( ) Don't know ( )

98. What is the source of water used in your home?
Cistern ( ) Well / *Cacimba* ( ) River/lake/canal ( ) Other ( ) Which one:________ Don't know ( )

99. What water do you use to drink?
Untreated water ( ) Treated with chlorine ( ) Boiled ( ) Filtered ( ) Don't know ( )

100. What water do you use to cook?
Untreated water ( ) Treated with chlorine ( ) Boiled ( ) Filtered ( ) Don't know ( )

101. Where do you store the water to drink?
Barrel ( ) Bottles ( ) Bucket/bown with lid ( ) Bucket/bown without lid ( ) Filter ( )
Other ( ) Which one: _____________________ Don't know ( )

102. Do you have chlorine at home? Yes ( ) No ( ) Don't know ( )
If YES, can you show me? Viewed ( ) Not viewed ( )
If YES, how did you get it? Received from the Health Unit ( ) Received from CHW ( ) Purchased ( ) Other ( ) Which one:______________ Don't Know ( )

103. Does your house have electricity?
Yes, public ( ) Yes, from generator ( ) Yes, other ( ) which one ________ No ( )

104. Does your house have:

105.

	Yes	No
Vertical refrigerator		
Horizontal refrigerator		
Stove		
Microwave		
Television		
Radio/ Stereo		
Cell phone/telephone		

106. Where does your household trash goes?
Collected ( ) Lying outside the courtyard of the house ( ) Lying in the courtyard of the house ( )
Burried ( ) Burned ( ) Other ( ) Which one: _______

107. Do you have mosquito bednets at home? Yes ( ) No ( )
If YES, how many: _______
If YES, can you show me? Viewed ( ) How many: __ Not viewed ( ) There is no mosquito bednet ( )

108. Check if bednets are set over the beds/mattresses?
Yes ( ) No ( ) Could not verify ( ) There is no bednet ( )

109. Who in the house slept under the mosquito bednet last night?
Pregnant woman: Yes ( ) No ( )
Children under five: Yes ( ) No ( )
Mother: Yes ( ) No ( )
Others: Yes ( ) No ( ) Who:____
There is no mosquito bednet ( )

DATA ABOUT CHW NA ( )

110. Has a community health worker ever visited your home?
Yes ( ) No ( ) Don't know ( ) Don't remember ( )
If YES, how many times has she/he come in the last 6 months ____ Don't remember ( )

111. When was the last time the CHW came to your house?
In the last month ( ) 1 to 3 months ago ( ) 3 to 6 months ago ( ) More than 6 months ago ( )
Don't remember ( )

112. What is the name of the CHW who visits your home ____________ Don't know ( ) Don't remember ( )

113. Do you know where the CHW (name of the CWH) lives? Yes ( ) No ( )

114. When the CHW comes to visit your house, what does he talk about? (do not read the alternatives, but encourage the mother to remember)
Water treatment Yes ( ) Not mentioned ( )
Use of mosquito bednets: Yes ( ) Not mentioned ( )
Pregnancy care: Yes ( ) Not mentioned ( )
Breastfeeding: Yes ( ) Not mentioned ( )
Child vaccination: Yes ( ) Not mentioned ( )
Other subject ( ) Which: ______________________

115. What would you say about CHWs? Would you like to say something about how CHW's work could improve? Would you like to say anything else before the interview finishes?

____
____
____
____
____

POSSESSION OF HEALTH CARDS

116. Do you have PREGNANCY CARD FOR (child's name) pregnancy?
Yes ( ) No, I never received one ( ) I did, but I lost it ( ) Don't know ( )

117. Can you show me the card? Yes, viewed ( ) Not viewed ( )

118. Do you have a HEALTH CARD (or VACCINATION CARD) for (child's name)?
Yes ( ) No, I never received one ( ) I did, but I lost it ( ) Don't know ( )

119. Can you show me the card? Yes, viewed ( ) Not viewed ( )

PREGNANCY CARD DATA No card ( )

Write down only the data that is registered on the card:

120. Gestational age of the first prenatal visit? _____ weeks

121. Number of prenatal visits: ___ No information ( ) NA ( )

122. Weight measured in prenatal visits?
Yes, in all the visits ( ) Yes, but not in all the visits ( ) Yes, only in one visit ( )
No information ( )

123. Blood pressure measured in prenatal visits?
Yes, in all the visits ( ) Yes, but not in all the visits ( ) Yes, only in one visit ( )
No information ( )

124. Anti-tetanus vaccine performed during pregnancy?
Yes, 1 dose ( ) Yes, 2 doses ( ) Yes, 3 doses ( ) No need, already vaccinated ( )
No information ( )

125. Did you have deworming (Albendazol) in pregnancy? Yes ( ) No information ( )

126. Did you receive medication to prevent malaria in pregnancy (Intermitent Preventive Treatment with Fansidar)?
Yes, 2 or more doses ( ) Yes, one dose No information ( )

127. Did you receive medication for anemia (iron salt and / or folic acid) during pregnancy?
Yes ( ) No information ( )

128. Any referral criteria recorded on the card? Yes ( ) No ( )
If YES, specify the problem that justifies referral to hospital: ____________________________

129. Registered postpartum visit? Yes ( ) No ( )

130. Any illness after childbirth? Yes ( ) No ( ) No information ( ) NA ( )

CHILD HEALTH CARD DATA   No card ( )

Write down only the data that is registered on the card:

131. Date of birth: ___/___/____

132. Place of childbirth:
At home ( )   In the Health Unit ( )   No information ( )

133. Type of delivery:
Vaginal ( )   Caesarean ( )   No information ( )

134. Birth weight: _____ grams   No information ( )

135. Record the date of application of each vaccine:

**BCG**<br>Performed ( )<br>No information ( )<br>Date:___/___/____	**POLIO ZERO**<br>Performed ( )<br>No information ( )<br>Date:___/___/____
**POLIO 1**<br>Performed ( )<br>No information ( )<br>Date:___/___/____	**PENTA 1**<br>Performed ( )<br>No information ( )<br>Date:___/___/____
**POLIO 2**<br>Performed ( )<br>No information ( )<br>Date:___/___/____	**PENTA 2**<br>Performed ( )<br>No information ( )<br>Date:___/___/____
**POLIO 3**<br>Performed ( )<br>No information ( )<br>Date:___/___/____	**PENTA 3**<br>Performed ( )<br>No information ( )<br>Date:___/___/____
**MEASLES**<br>Performed ( )<br>No information ( )<br>Date:___/___/____	**YELLOW FEVER**<br>Performed ( )<br>No information ( )<br>Date:___/___/____

136. Record Vitamin A doses administration and dates:

Vitamin A 1st dose	Performed ( )<br>No information ( )<br>Date:___/___/____
Vitamin A 2nd dose	Performed ( )<br>No information ( )<br>Date:___/___/____

137. Record the date of delivery of the mosquito bednet:

Delivery of mosquito bednets	Performed ( )<br>No information ( )

	Date:___/___/____

138. If the child is aged 1 year or older, record deworming (Albendazole) doses administration and dates. NA ( )

Albendazole
Date:__/__/_____ No information ( )
Date:__/__/_____ No information ( )
Date:__/__/_____ No information ( )
Date:__/__/_____ No information ( )

139. Write down the age of the child when he/she was last weighted _____ months No information ( )

140. How many times is the weight recorded on the card? _____

Now you can thank the mother for her availability and attention.

Before you say goodbye:

- inform that the COORDINATOR will come to weigh and measure the child
- say she will receive a photo of her and her child for having participated in the survey

COORDINATOR: Calculate if the vaccines are updated according to the child's age.

Source of information: Card ( ) Mother( )

BCG	Yes ( ) No ( )
Polio	Yes ( ) No ( )
Pentavalent	Yes ( ) No ( )
Measles	Yes ( ) No ( )
Yellow Fever	Yes ( ) No ( )

Is the weight monitoring interval range suitable for the child's age? Yes ( ) No ( )

COORDINATOR

MEASURES OF CHILD'S WEIGHT AND LENGTH

Explain to the mother, "Now let's measure the weight and size of the child." Please, can you take his/her clothes off (child's name)?
The weight will be measured together with the mother on the scale.

	**Length**	**Mother's weight**	**Mother's weight with child**	**Child's weight**
**Measure 1**				
**Measure 2**				
**Mean**				

Does the child have edema in the lower limbs? ( ) Yes No ( )

Locate in growth curves:

	< p3	p3 a p15	p15 a p85	p85 a p97	> p97
Weight/height					
Weight /Age					
Altura/ Age					
BMI					
